# Class-Sensitive TPB-Guided Memory Refinement for Online Zero-Shot Anomaly Detection

**DOI:** 10.3390/s26113537

**Published:** 2026-06-03

**Authors:** Zhen Zhao, Fan Song, Xinyun Wang, Tianshun Yuan, Jiali Zhou

**Affiliations:** 1 School of Intelligent Manufacturing, Zhejiang Polytechnic University of Mechanical and Electrical Engineering, Hangzhou 310053, China; zhaozhen@zime.edu.cn; 2 College of Mathematical Sciences, Zhejiang University of Technology, Hangzhou 310023, China; 19016952928@163.com (F.S.); 2112109006@zjut.edu.cn (X.W.); tianshun_yuan@163.com (T.Y.)

**Keywords:** zero-shot anomaly detection, vision–language models, CLIP, online memory refinement, test-time adaptation

## Abstract

Zero-shot anomaly detection is attractive for industrial inspection, where target-domain training data are often unavailable for newly introduced products. Recent CLIP-based methods have demonstrated promising generalization, and online memory mechanisms can further improve adaptability by incorporating incoming test samples. However, unreliable or ambiguous evidence may be incorporated during online memory updates, which can degrade subsequent predictions, especially for weak or visually unstable categories. In this work, we propose TSMR, a lightweight extension of RareCLIP for online zero-shot anomaly detection. Rather than modifying the backbone or redesigning the anomaly scoring pipeline, TSMR improves the reliability of test-time memory evolution through a class-sensitive selective update strategy. Specifically, it combines a confidence quantile gate, a text-prior-based reliability check, and weak-class selective activation to derive a frame-level memory-update decision during online inference. Experiments on VisA and MVTec AD show that TSMR achieves clear improvements on VisA while maintaining competitive performance on MVTec AD. Under the online protocol, TSMR improves the reproduced RareCLIP baseline on VisA from 94.4% to 95.1% in image-level AUROC, from 98.8% to 98.9% in pixel-level AUROC, and from 93.5% to 94.0% in PRO. On MVTec AD, TSMR achieves 98.0% image-level AUROC, 97.6% pixel-level AUROC, and 93.6% PRO, remaining competitive with the strong RareCLIP baseline. Object-wise and seed-wise analyses further indicate that selective memory refinement is particularly beneficial for selected weak categories and remains stable across different online evaluation orders. These results suggest that reliable online memory evolution is an effective direction for CLIP-based zero-shot anomaly detection.

## 1. Introduction

Anomaly detection is a fundamental task in machine learning and computer vision, with broad applications in medical diagnosis, video surveillance, network security, remote sensing, and financial risk monitoring [1]. In these scenarios, the goal is to identify rare or abnormal patterns that deviate from expected normal behavior. Among these applications, industrial anomaly detection plays an important role in modern manufacturing and quality inspection, where reliable defect detection is required under diverse deployment conditions [2,3]. Traditional unsupervised anomaly detection methods usually rely on a set of normal training images to learn category-specific normal patterns. Although these methods have achieved strong performance on standard benchmarks, their dependence on target-domain normal data limits their applicability to newly introduced products. In practical industrial scenarios, new categories may need to be inspected immediately, while collecting and annotating sufficient normal samples for each product is often expensive or impractical. This motivates zero-shot anomaly detection (ZSAD), which aims to detect anomalies in unseen categories without using target-domain training samples [4].

With the strong generalization ability of vision–language models, CLIP-based ZSAD has become an active research direction. Recent surveys also highlight that Transformers and foundation models are reshaping visual anomaly detection by improving contextual modeling, transferability, and zero-/few-shot generalization [5,6]. Most existing methods compare test images with predefined or learned normal/abnormal textual prompts, or improve visual–text alignment through prompt learning, anomaly-aware semantic modeling, context adaptation, and local feature enhancement [7,8,9,10,11,12,13]. These methods have achieved promising zero-shot anomaly classification and localization performance. However, they usually process each test image independently and therefore underuse the relationships among test images in real industrial inspection streams.

To exploit such test-time relationships, recent studies have explored batch and online zero-shot anomaly detection. Batch methods such as MuSc [14] use mutual scoring among multiple unlabeled test images, showing that inter-image relationships can provide useful anomaly cues. However, batch-level inference usually requires access to multiple test images at the same time and may introduce additional computational cost. More recently, RareCLIP [15] introduced an online zero-shot anomaly detection paradigm, where test images are processed sequentially and the memory bank evolves during inference. By combining CLIP with dynamic rarity modeling, RareCLIP exploits the observation that normal patches tend to appear consistently over time, whereas abnormal patches are rare and variable. It therefore maintains online patch memories to estimate rarity during sequential inference.

Despite this progress, RareCLIP-style online memory evolution raises a new reliability issue. Unlike conventional memory-bank methods, where memory is constructed from clean normal training samples, the online memory in RareCLIP is updated from unlabeled test streams. Therefore, the evidence used for memory updating may involve normal regions, defective regions, ambiguous visual patterns, or category-specific appearance variations. Once unreliable evidence is incorporated into the memory, it can affect subsequent predictions and lead to error accumulation. This issue is particularly important for weak or visually unstable categories, where anomaly evidence is subtle and the original update rule may not always provide reliable memory evolution. While existing online or test-time methods mainly focus on nominal model adaptation, multi-image inference, or anomaly scoring, we focus on the reliability control of the memory evolution process itself.

Motivated by this observation, we propose TSMR, a TPB-guided Selective Memory Refinement strategy for online zero-shot anomaly detection. TSMR is designed as a lightweight extension of RareCLIP. Instead of modifying the CLIP backbone or redesigning the anomaly scoring pipeline, it focuses on regulating the online memory update process. Specifically, TSMR combines confidence-based self-calibration, text-prior-based reliability assessment, and weak-class selective activation to derive a frame-level memory-update decision during sequential inference. In this way, TSMR preserves the efficiency and transferability of RareCLIP while improving the reliability of online memory evolution for difficult categories.

Our main contributions are summarized as follows:

- We identify the reliability of online memory updating as a key issue in RareCLIP-style online zero-shot anomaly detection. Since online memory is updated from unlabeled test streams rather than clean normal training data, unreliable or ambiguous update evidence may affect subsequent predictions, especially for weak or visually unstable categories.
- We propose TSMR, a class-sensitive text-prior-based (TPB) selective memory refinement strategy for online memory evolution. Different from uniform online updating, TSMR integrates confidence-based self-calibration, text-prior-based reliability assessment, and weak-class selective activation to derive a frame-level memory-update decision during sequential inference, without modifying the backbone encoder or the anomaly scoring pipeline.
- We provide object-wise and seed-wise analyses on VisA and MVTec AD. The results show that TSMR improves the reproduced RareCLIP baseline on VisA, with gains mainly concentrated on selected weak categories, while maintaining competitive performance on MVTec AD.

The remainder of this paper is organized as follows. Section 2 reviews related work on CLIP-based zero-shot anomaly detection and online memory adaptation. Section 3 presents the proposed TSMR framework. Section 4 describes the experimental settings, quantitative and qualitative results, ablation studies, and cost analysis. Section 5 concludes the paper and discusses future directions.

## 2. Related Work

### 2.1. Zero-Shot Anomaly Detection with Vision–Language Models

Zero-shot anomaly detection aims to identify anomalies in unseen categories without using target-domain training data, making it particularly attractive for practical industrial inspection. Benefiting from the strong generalization capability of large-scale vision–language models, CLIP-based methods have become a major paradigm in this setting. WinCLIP [7] is a representative early work that introduces CLIP into zero-/few-shot anomaly classification and segmentation through prompt engineering and multi-scale window aggregation. Subsequent studies extend this line from multiple perspectives. AnomalyCLIP [8] learns object-agnostic prompts for generalized abnormality recognition, while AA-CLIP [9] enhances anomaly discrimination by explicitly modeling normal and abnormal semantics. AdaCLIP [10] and VCP-CLIP [11] further improve CLIP-based zero-shot anomaly detection through hybrid learnable prompts and visual-context prompting, respectively. Other methods, such as PromptAD [16], PA-CLIP [17], SOWA [18], LECLIP [12], AF-CLIP [13], FE-CLIP [19], AdaptCLIP [20], DyC-CLIP [21], FADE [22], GenCLIP [23], and IAP-AS [24], explore prompt optimization, pseudo-anomaly awareness, hierarchical feature modeling, local enhancement, anomaly-focused adaptation, frequency-aware enhancement, adaptive CLIP tuning, dynamic context prompting, large vision–language models, and image-aware prompt generation. Recent hybrid foundation-model approaches further combine CLIP with complementary visual encoders such as DINOv2 to improve both semantic generalization and fine-grained structural perception [25]. Despite these advances, most existing methods are mainly driven by external priors such as textual prompts, prompt-tuned embeddings, or auxiliary reference features, while the inference procedure itself remains less explored.

### 2.2. Online Memory and Test-Time Adaptation

Beyond static zero-shot inference, recent studies have begun to consider online anomaly detection and test-time adaptation under evolving test distributions. Online-InReaCh [26] proposes a fully unsupervised online method that dynamically maintains a nominal patch model for anomaly detection under non-stationary image distributions. MuSc [14] introduces mutual scoring over unlabeled test images, showing that inference–time interactions among test samples can provide additional useful cues for zero-shot anomaly classification and segmentation. Recent work on consistent anomalies further shows that repeatedly occurring anomalous patterns in unlabeled test images can challenge zero-shot anomaly detection, highlighting the need for reliable test-time reasoning and update control [27]. More recently, RareCLIP [15] incorporates a rarity-aware online memory mechanism into CLIP-based industrial anomaly detection, enabling the memory bank to evolve sequentially during inference.

However, online memory updating also introduces a new source of error accumulation. Since memory is updated from incoming test samples, unreliable or ambiguous evidence may be incorporated during online memory updates and subsequently affect later predictions. This issue is particularly critical for weak or visually unstable categories, where anomaly evidence is subtle and update reliability is harder to estimate. Although online adaptation has shown clear potential, the reliability control of memory updates remains underexplored.

Different from these studies, our work focuses on the reliability control of online memory updating in RareCLIP-style online zero-shot anomaly detection. Online-InReaCh maintains a nominal patch model for non-stationary image streams, while MuSc exploits mutual scoring among multiple unlabeled test images. RareCLIP introduces rarity-aware online memory evolution, but its memory update is still applied in a relatively uniform manner across categories. In contrast, TSMR does not redesign the anomaly scoring function or require batch-level interactions among test images. Instead, it introduces a class-sensitive selective update rule that uses confidence calibration and text-prior-based evidence to regulate whether the online memory should be refined for weak categories.

### 2.3. Rarity-Aware and Multi-Cue Anomaly Reasoning

Besides semantic deviation, anomalies are often characterized by inconsistency with surrounding or repetitive local patterns, such as local structural disruption, abnormal connectivity, or rare patch configurations. Several recent methods have explored complementary anomaly cues beyond pure patch-text alignment. RareCLIP [15] highlights the importance of rarity-aware reasoning in online zero-shot industrial anomaly detection. ClipSAM [28] strengthens anomaly localization by combining CLIP and SAM, while CLIPFUSION [29] integrates CLIP with diffusion-based priors. Methods such as SOWA [18], LECLIP [12], AF-CLIP [13], and FE-CLIP [19] also improve anomaly perception through hierarchical modeling, local enhancement, anomaly-focused adaptation, and frequency-aware cues. Recent foundation-model-based methods further exploit DINOv2 or multimodal large language models to enhance structural perception and anomaly reasoning [30,31,32]. Although these methods demonstrate the value of richer anomaly evidence, such cues are still mainly incorporated at the feature level or the scoring level. Their role in guiding more reliable inference–time decisions, particularly under online memory updating conditions, remains underexplored.

## 3. Method

### 3.1. Overview

Here, we study online zero-shot anomaly detection for industrial images, where no target-domain training samples are used during downstream deployment. Given a test image $x_{t}$, the objective is to predict both a pixel-level anomaly map $A_{\mathrm{pix}}$ and an image-level anomaly score $S_{\mathrm{img}}$, while allowing the model to refine its memory state sequentially during inference.

**Figure 1:**
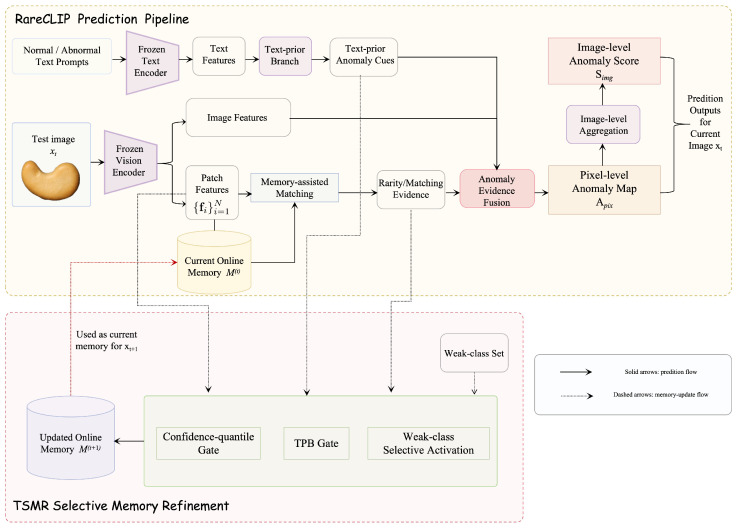
Overall framework of TSMR.

We propose TSMR, a TPB-guided Selective Memory Refinement framework built upon the reproduced RareCLIP baseline. As illustrated in Figure 1, TSMR keeps the original visual–text encoder and anomaly scoring pipeline unchanged, and instead improves the reliability of online memory evolution through a selective update strategy.

We explicitly distinguish the prediction outputs from the memory-update output. For the current test image $x_{t}$, the reproduced RareCLIP prediction pipeline uses the current memory state $M^{\left(t\right)}$ to produce the pixel-level anomaly map $A_{\mathrm{pix}}$ and the image-level anomaly score $S_{\mathrm{img}}$. The memory-assisted matching module provides reference-matching and rarity-related evidence for anomaly map construction and image-level aggregation, while the text-prior branch provides complementary semantic anomaly cues. After the prediction for $x_{t}$ is obtained, TSMR regulates the transition from $M^{\left(t\right)}$ to $M^{\left(t+1\right)}$. Therefore, the selective memory refinement module is not the anomaly prediction output for the current image; instead, it updates the online memory and affects subsequent predictions through the refined memory state.

Specifically, TSMR consists of four coupled components: (i) the reproduced RareCLIP anomaly reasoning pipeline, which extracts patch-level anomaly evidence and produces image-level and pixel-level predictions; (ii) a confidence quantile gate, which estimates the frame-level reliability of the current update; (iii) a text-prior-based (TPB) reliability check, which uses textual anomaly evidence to avoid unreliable memory refinement; and (iv) a weak-class selective activation mechanism, which enables the proposed refinement only for selected difficult categories to avoid unnecessary interference on already stable classes.

Different from retraining-based adaptation methods, TSMR does not introduce a new optimization stage or alter the backbone architecture. Instead, it acts as a lightweight plug-in refinement rule at test-time. In the online setting, patch-level confidence and TPB evidence are used to derive a frame-level memory-update decision. When the update condition is satisfied for an activated weak category, the online memory is refined from $M^{\left(t\right)}$ to $M^{\left(t+1\right)}$ for subsequent inference. In this way, TSMR improves the stability of test-time updating while preserving the efficiency and transferability of the original zero-shot framework. At inference time, anomaly maps and image-level scores are still computed by the original RareCLIP scoring pipeline, whereas the proposed refinement affects prediction indirectly through a more reliable memory state. The main symbols and their definitions are summarized in Table 1.

### 3.2. Baseline Online Memory Update

Let ${\left\{f_{i}\right\}}_{i=1}^{N}$ denote the patch-level visual features extracted from the current test image, where *N* is the number of patches. In the baseline RareCLIP framework, the online memory is updated sequentially during inference. Denoting the memory state before processing the current image by $M^{\left(t\right)}$, the baseline update can be abstractly written as

(1) $$M^{\left(t+1\right)}=U\left(M^{\left(t\right)},{\left\{f_{i}\right\}}_{i=1}^{N}\right),$$

where U(·) denotes the original RareCLIP memory-update operator.

This update enables the memory to evolve with the incoming test stream and improves the adaptability of online zero-shot anomaly detection. However, the baseline update is applied to each incoming frame in a relatively uniform manner. Since the test stream is unlabeled, the current frame may contain normal regions, defective regions, ambiguous visual evidence, or category-specific appearance variations. If unreliable evidence is repeatedly incorporated into the memory, it may affect subsequent predictions. This issue is more evident for weak or visually unstable categories, where the reliability of online memory evolution is harder to estimate.

Based on this observation, our goal is not to redesign the anomaly scoring pipeline, but to regulate whether the original memory-update operator U(·) should be applied to the current frame. In other words, TSMR introduces a frame-level reliability gate before the baseline memory update, while keeping the backbone encoder and the RareCLIP scoring functions unchanged.

### 3.3. TPB-Guided Selective Memory Refinement

**Figure 2:**
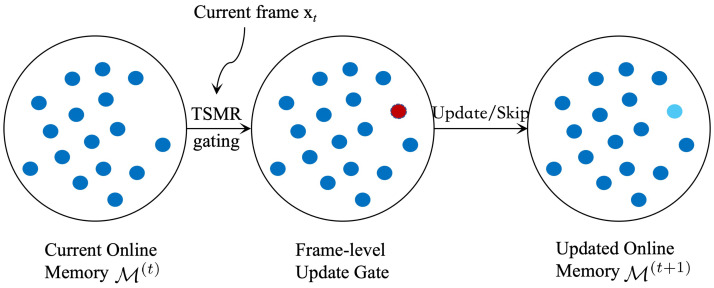
Illustration of the TPB-guided frame-level selective memory refinement mechanism. Red denotes incoming information; light blue denotes updated memory.

Figure 2 illustrates the core idea of the proposed frame-level selective memory refinement mechanism. Given the current online memory state $M^{\left(t\right)}$ and the incoming test frame $x_{t}$, TSMR estimates whether the current frame is reliable enough for memory refinement. Specifically, patch-level matching confidence and text-prior-based evidence are first computed from $x_{t}$ and then aggregated into frame-level gates. These gates produce the final update decision $G_{t}^{\mathrm{patch}}$, which determines whether the original RareCLIP memory-update operator should be applied to the current frame. If the gate is satisfied, the memory is updated from $M^{\left(t\right)}$ to $M^{\left(t+1\right)}$ using the original update operator; otherwise, the memory state remains unchanged. Therefore, TSMR regulates the frame-level transition of the online memory, rather than sparsely writing individual selected patches into memory.

#### 3.3.1. Reference Confidence Quantile Gate

We first use the memory-assisted matching confidence to estimate whether the current frame is reliable for online memory refinement. Let $m_{i}$ denote the reference confidence score of patch $f_{i}$, which is obtained from the memory-assisted matching branch. We compute the frame-level mean reference confidence as

(2) $${\bar{m}}_{t}=\frac{1}{N}\sum_{i=1}^{N}m_{i}.$$

To avoid using a fixed confidence threshold across different online streams, we maintain a history set H of previous frame-level confidence values. Let $p_{q}$ denote the quantile parameter and $N_{\min}$ denote the minimum number of historical frames required to activate this gate. The reference confidence quantile gate is defined as

(3) $$G_{\mathrm{cq}}=\left\{\begin{array}{cc} 1, & |H|<N_{\min},\\ I\left({\bar{m}}_{t}\geq \mathrm{Percentile}\left(H,p_{q}\right)\right), & \left|H\right|\geq N_{\min}, \end{array}\right.$$

where I(·) is the indicator function. When the history is not sufficiently long, this gate is not used to block memory updating. Otherwise, the current frame is allowed to pass the confidence gate only when its mean reference confidence is not lower than the historical quantile threshold. After the gate is evaluated for the current frame, ${\bar{m}}_{t}$ is appended to H for subsequent online calibration. This design provides a self-calibrated frame-level confidence check for online memory refinement.

#### 3.3.2. Text-Prior-Based Frame Consensus Gate

Confidence alone is not sufficient, because a frame may still contain suspicious regions even when the visual features can be confidently matched to the memory. Therefore, we further introduce a text-prior-based (TPB) frame consensus gate. This gate combines memory-assisted reference evidence and text-prior anomaly evidence to judge whether the current frame is suitable for memory refinement.

Let ${\hat{r}}_{i}$ denote the reference consistency score of patch *i* from the memory-assisted matching branch, and let $p_{i}^{\mathrm{abn}}$ denote the text-prior abnormal probability of patch *i*. In our implementation, $m_{i}$ and ${\hat{r}}_{i}$ are both derived from the patch-level reference confidence scores produced by the memory-assisted matching branch. We use $m_{i}$ to compute the frame-level mean confidence for quantile calibration, and ${\hat{r}}_{i}$ to denote the patch-level reference consistency evidence used in the TPB frame consensus gate.

A patch is regarded as reliable for memory refinement when it has sufficiently high reference consistency and sufficiently low text-prior abnormal probability:

(4) $$a_{i}=I\left({\hat{r}}_{i}>\tau_{r}\right)\;I\left(p_{i}^{\mathrm{abn}}<\tau_{t}\right),$$

where $\tau_{r}$ and $\tau_{t}$ are the reference consistency threshold and the text-prior abnormality threshold, respectively. We then compute the frame-level reliable ratio as

(5) $$\rho_{\mathrm{tpb}}=\frac{1}{N}\sum_{i=1}^{N}a_{i}.$$

The TPB frame consensus gate is defined as

(6) $$G_{\mathrm{tpb}}=I\left(\rho_{\mathrm{tpb}}\geq \tau_{f}\right),$$

where $\tau_{f}$ is the frame-level fraction threshold. In our implementation, we set $\tau_{f}=0.50$. Therefore, the current frame passes the TPB gate only when at least half of its patches satisfy both the reference consistency condition ${\hat{r}}_{i}>\tau_{r}$ and the text-prior normality condition $p_{i}^{\mathrm{abn}}<\tau_{t}$. If this ratio is lower than $\tau_{f}$, the current frame is regarded as unreliable for memory refinement and is not used to update the online memory for activated weak categories. The values of $\tau_{r}$, $\tau_{t}$, and $\tau_{f}$ are fixed before the final online evaluation, and the selection procedure is described in the implementation details.

#### 3.3.3. Weak-Class Selective Activation

A key observation from our pilot experiments is that not all categories benefit equally from additional memory filtering. For many categories, the baseline RareCLIP update is already sufficiently stable, and extra gating may bring little benefit or even introduce unnecessary interference. Therefore, instead of applying the proposed refinement to all categories uniformly, we activate it only for a selected weak-class subset.

Let *y* denote the category identity of the current test sample, and let $\Omega_{w}$ denote the activated weak-class set. The final frame-level patch-memory update gate is defined as

(7) $$G_{t}^{\mathrm{patch}}=\left\{\begin{array}{cc} G_{\mathrm{cq}}\cdot G_{\mathrm{tpb}}, & y\in \Omega_{w},\\ 1, & y\notin \Omega_{w}. \end{array}\right.$$

For categories outside the weak-class set, $G_{t}^{\mathrm{patch}}=1$, and TSMR reduces to the original RareCLIP updating behavior. For activated weak categories, memory refinement is performed only when both the reference confidence quantile gate and the TPB frame consensus gate are satisfied.

The resulting online memory update is written as

(8) $$M^{\left(t+1\right)}=\left\{\begin{array}{cc} U\left(M^{\left(t\right)},{\left\{f_{i}\right\}}_{i=1}^{N}\right), & G_{t}^{\mathrm{patch}}=1,\\ M^{\left(t\right)}, & G_{t}^{\mathrm{patch}}=0. \end{array}\right.$$

Thus, TSMR controls whether the original RareCLIP memory-update operator U(·) is applied to the current frame. When the gate is activated, the current frame updates the online memory using the original update operator; otherwise, the memory state remains unchanged for subsequent inference. This frame-level gated refinement is consistent with the memory layout of the reproduced RareCLIP implementation and avoids the misleading interpretation that individual selected patches are sparsely written into memory.

### 3.4. Inference and Scoring

TSMR does not modify the backbone encoder, the anomaly map construction function $\Phi_{\mathrm{pix}}\left(\cdot \right)$, or the image-level scoring function $\Phi_{\mathrm{img}}\left(\cdot \right)$ of RareCLIP. For each incoming test image, prediction is performed using the current memory state $M^{\left(t\right)}$ before memory updating. Specifically, the current memory and visual features are first used by the reproduced RareCLIP pipeline to generate the pixel-level anomaly map and the image-level anomaly score. After the prediction is completed, the proposed selective update rule regulates whether the memory should be refined to $M^{\left(t+1\right)}$ for subsequent inference.

Formally, given the current memory state $M^{\left(t\right)}$ and the patch-level visual features ${\left\{f_{i}\right\}}_{i=1}^{N}$ extracted from the input image, the baseline RareCLIP inference pipeline produces a pixel-level anomaly map

(9) $$A_{\mathrm{pix}}=\Phi_{\mathrm{pix}}\left(x,{\left\{f_{i}\right\}}_{i=1}^{N},M^{\left(t\right)}\right),$$

where $\Phi_{\mathrm{pix}}\left(\cdot \right)$ denotes the baseline anomaly map construction function. Based on the resulting anomaly map, the image-level anomaly score is computed as

(10) $$S_{\mathrm{img}}=\Phi_{\mathrm{img}}\left(A_{\mathrm{pix}}\right),$$

where $\Phi_{\mathrm{img}}\left(\cdot \right)$ denotes the baseline image-level aggregation function.

After the prediction for the current image is obtained, TSMR selectively updates the memory according to the gated update rule, producing $M^{\left(t+1\right)}$ for subsequent inference. In the offline setting, memory updating is disabled and the evaluation is deterministic. In the online setting, the proposed selective refinement affects future predictions through the updated memory state. In this way, TSMR remains a lightweight plug-in strategy that preserves the original RareCLIP inference pipeline while improving the reliability of online memory evolution on selected weak categories.

## 4. Experiments

### 4.1. Datasets

VisA [33] is a challenging industrial anomaly detection benchmark that has been widely adopted for evaluating zero-shot and test-time adaptive anomaly detection methods. It contains 12 product categories with both image-level anomaly labels and pixel-level ground-truth masks for anomalous samples. Compared with more canonical industrial benchmarks, VisA exhibits larger intra-class appearance variations, more cluttered backgrounds, and more diverse anomaly patterns. These characteristics make it a suitable testbed for evaluating whether a method can maintain robust anomaly discrimination and localization performance under visually complex conditions.

MVTec AD [34] is one of the most established benchmarks for industrial anomaly detection. It contains 15 categories, including 10 object categories and 5 texture categories, with image-level labels and pixel-level ground-truth masks for anomalous samples. The anomaly types in MVTec AD cover a variety of industrial defects, such as scratches, cracks, contamination, holes, and structural irregularities. Owing to its relatively standardized acquisition setup and wide adoption in the literature, MVTec AD serves as a strong reference benchmark for evaluating both anomaly classification and fine-grained localization quality.

In this work, both datasets are used only for evaluation under the zero-shot setting. No target-domain training images are used to train or fine-tune the backbone model or the proposed TSMR module. For each dataset, we follow the official category definitions and use the image-level labels and pixel-level ground-truth masks for computing I-AUC, P-AUC, and PRO.

For online evaluation, each object category is treated as an independent sequential test stream. The online memory is initialized before processing a category, updated sequentially as test images arrive, and reset before evaluating the next category. Thus, the memory evolution is category-specific and does not mix information across different object categories. Within each category, different random seeds correspond to different test-image orders, which are used to evaluate the stability of online memory updating.

VisA is used for the main analysis of the proposed TSMR refinement, including object-wise comparison, seed-wise stability analysis, weak-class activation, and hyperparameter sensitivity. MVTec AD is used as an additional widely adopted benchmark to examine whether the proposed memory refinement remains competitive under the same zero-shot and online evaluation protocol. This usage reflects the goal of our method: improving the reliability of RareCLIP-style online memory evolution, especially on visually challenging categories, while preserving the performance and transferability of the original zero-shot framework.

### 4.2. Evaluation Metrics

Image-level AUROC (I-AUC) [35] measures the ability of a method to distinguish normal images from anomalous images according to the predicted image-level anomaly scores. As a threshold-independent metric, it is widely used to assess image-level anomaly classification performance.

Pixel-level AUROC (P-AUC) [34,35] evaluates anomaly localization quality by comparing the predicted anomaly map with the ground-truth pixel-level annotation. It reflects how well the predicted anomaly responses separate anomalous pixels from normal pixels over all possible thresholds.

Per-Region Overlap (PRO) [34] measures the localization quality of anomaly maps from a region-aware perspective. Compared with pixel-level AUROC, PRO places more emphasis on whether anomalous regions are correctly covered as coherent defect regions, and is therefore widely used to evaluate the practical usefulness of anomaly localization results in industrial inspection scenarios.

Higher values indicate better performance for all metrics.

### 4.3. Implementation Details

Our model is implemented in PyTorch 1.13.1 with Python 3.10 and torchvision 0.14.1, using CUDA 11.6 on top of the reproduced RareCLIP framework. Since TSMR is designed as a lightweight test-time refinement strategy, we do not introduce an additional training stage beyond the pretrained baseline model. All comparisons are conducted under the same reproduced backbone, preprocessing pipeline, and evaluation protocol to ensure fairness. All experiments are conducted on an NVIDIA GeForce RTX 3090 GPU (NVIDIA Corporation, Santa Clara, CA, USA).

We report both offline and online results. In the offline setting, test-time memory updating is disabled and the evaluation is deterministic. In the online setting, memory is updated sequentially during inference, and the results may depend on the order of incoming test samples. Therefore, we use five seeds, i.e., seeds 0–4, to evaluate the stability of online memory updating under different test orders. This five-seed setting follows the reproduced RareCLIP online evaluation protocol and provides a practical estimate of order-induced variation without changing the pretrained model or introducing additional training. For each seed, the random seed is fixed, and the test images are shuffled within each object category using the same seed. The categories are evaluated one by one in a fixed category order, and the online memory bank is reset before evaluating each category.

For the object-wise analysis, we evaluate each object category separately rather than pooling all categories together. For each category, I-AUC is computed using image-level normal/anomalous labels, while P-AUC and PRO are computed using pixel-level ground-truth masks for anomalous regions. The object-wise results are obtained by averaging the corresponding category-level metrics over the five seeds. The mean row is the unweighted average over all categories. For the seed-wise analysis, each seed corresponds to one complete multi-category online evaluation run. For each seed, we first compute the category-wise metrics and then report the macro-average over all categories. The mean and standard deviation are computed across the five seeds.

For the proposed TSMR gates, we set $p_{q}=0.10$ for the reference confidence quantile gate and use $N_{\min}=200$ as the minimum history length before activating this gate. The confidence quantile parameter $p_{q}$ is analyzed separately in the sensitivity study. For the TPB frame consensus gate, three thresholds are involved: $\tau_{r}$ for the reference consistency condition, $\tau_{t}$ for the text-prior abnormality condition, and $\tau_{f}$ for the frame-level reliable ratio condition. We determine these thresholds using a reproducible development protocol on VisA with online seed 0. Specifically, $\tau_{r}$ is fixed to 0.35 as a moderate reference consistency threshold on the normalized patch-level matching scores. We then perform a one-dimensional search for $\tau_{t}\in \left\{0.38,0.42,0.46,0.50\right\}$ with $\tau_{f}=0.50$, and for $\tau_{f}\in \left\{0.30,0.50,0.70\right\}$ with $\tau_{t}=0.42$, using macro-average I-AUC on VisA as the selection criterion. The selected setting is $\left(\tau_{r},\tau_{t},\tau_{f}\right)=\left(0.35,0.42,0.50\right)$, which is fixed for all five-seed online evaluations reported in the main tables. Here, $\tau_{f}=0.50$ means that a frame passes the TPB gate only when at least half of its patches satisfy both the reference consistency condition and the text-prior normality condition. No per-seed tuning is performed in the reported results.

The same TPB thresholds are transferred to MVTec AD without additional threshold tuning. The activated weak-class set is determined separately for each dataset before the final five-seed evaluation; specifically, we use $\Omega_{w}^{\mathrm{VisA}}=\left\{\mathrm{capsules},\mathrm{macaroni}2\right\}$ for VisA and $\Omega_{w}^{\mathrm{MVTec}}=\left\{\mathrm{metal}\text{\_}\mathrm{nut}\right\}$ for MVTec AD. For categories outside the activated weak-class set, TSMR follows the original RareCLIP memory-updating behavior. For fair comparison, under the same seed, the reproduced RareCLIP baseline and TSMR use the same pretrained backbone, preprocessing pipeline, per-category test order, and metric implementation. The only difference is the online memory-update rule.

### 4.4. Main Results

We first compare the proposed method with representative zero-shot anomaly detection methods on the VisA and MVTec AD benchmarks, as summarized in Table 2. Since our method is built upon RareCLIP and mainly modifies the test-time memory refinement strategy, we group the compared methods by testing paradigm and report both datasets in a unified manner. It should be noted that MuSc* and TSMR follow different test-time inference paradigms. MuSc* leverages mutual scoring across multiple unlabeled test images and therefore belongs to a batch or multi-image inference setting. In contrast, TSMR follows the causal online protocol of RareCLIP, where test images are processed sequentially and each prediction uses only the current image and the memory accumulated from previous samples. Therefore, the comparison with MuSc* is included as a reference under different test-time information assumptions, rather than as a strictly identical online baseline comparison. In the offline setting, memory updating is disabled; therefore, the offline results mainly reflect the reproduced zero-shot scoring pipeline rather than the online memory-refinement effect of TSMR. Under the online protocol, where memory evolution is enabled, TSMR improves the reproduced RareCLIP baseline on VisA from 94.4% to 95.1% in I-AUC, from 98.8% to 98.9% in P-AUC, and from 93.5% to 94.0% in PRO. On MVTec AD, TSMR maintains the same I-AUC as the reproduced RareCLIP baseline and slightly improves P-AUC and PRO. These results indicate that the proposed selective memory refinement is most beneficial for visually challenging VisA categories, while preserving competitive performance on MVTec AD under the same online protocol.

To further remove possible confounding factors caused by implementation details, we conduct a fair in-house comparison under the same reproduced RareCLIP framework. Table 3 presents the object-wise comparison between the reproduced RareCLIP baseline and the proposed method on VisA under the online setting. Each entry is the average over five seeds for the corresponding object category, and the mean row is the unweighted average over all categories. Compared with the reproduced baseline, our method achieves a better overall trade-off across the three core metrics while keeping the same backbone, training protocol, and evaluation pipeline. Although the average gains are naturally limited by the already strong baseline, the object-wise results reveal a clearer and more informative improvement pattern than the mean values alone.

More specifically, the benefits of the proposed method are concentrated on a subset of challenging categories rather than being uniformly distributed over the entire dataset. The most noticeable improvement is observed on macaroni2, and a clear gain is also obtained on the activated weak category capsules. This trend is consistent with our motivation that TPB-guided selective memory refinement is particularly helpful for categories whose anomaly evidence is relatively subtle or unstable under the original online update mechanism. At the same time, slight negative transfer can still be observed on a few categories, indicating that the proposed refinement is not universally beneficial to every object category.

To examine whether the observed gains are stable across different online test orders, we further report the seed-wise comparison in Table 4. Each seed corresponds to a different within-category test order, and the reported value is the macro-average over all object categories under that seed. Following the reproduced RareCLIP online protocol, we use five seeds to estimate both the mean performance and the standard deviation caused by order sensitivity in online memory updating. The proposed method consistently outperforms the reproduced RareCLIP baseline across all five seeds on the three mean metrics, showing that the improvement is not caused by a particular test order or a single favorable run. This result is important because the online setting is inherently order-sensitive, and the seed-wise comparison confirms that the advantage of the proposed method remains stable under different evaluation orders.

Overall, Table 2, Table 3 and Table 4 support two main conclusions. Firstly, the proposed method consistently improves the reproduced RareCLIP baseline on VisA in a fair setting, while remaining competitive on MVTec AD. Secondly, the improvement mainly comes from more effective refinement on selected weak or difficult categories, rather than from a uniform gain across all classes. This observation further motivates the TSMR design analyzed in the following ablation study.

### 4.5. Ablation Study

We next analyze two key questions behind the final TSMR configuration: which weak-class subset should be activated, and how sensitive the method is to the patch gate quantile. The corresponding results are reported in Table 5 and Table 6.

#### 4.5.1. Effect of Weak-Class Subset Selection

To determine the activated weak-class set, we first perform pilot screening with single-class weak-only activation on VisA. The screening is used to identify candidate categories for ablation rather than to exhaustively search all possible subsets. Based on the pilot results and object-wise behavior, capsules and macaroni2 are selected as the main weak-class candidates, while pcb1 is included as an additional difficult candidate to examine whether enlarging the activated set further improves performance. Table 5 studies the effect of activating different candidate weak-class subsets. The results show that a broader activation set does not necessarily lead to better performance. In particular, activating all three candidate weak classes in A1 does not improve the final mean performance and is slightly inferior to the final configuration. By contrast, activating only capsules already yields a competitive result, while further including macaroni2 gives the best overall trade-off and is therefore adopted as the final setting.

It is worth noting that the margin between the tested subsets is not large. Therefore, the role of weak-class subset selection should not be interpreted as producing a dramatic mean-level improvement by itself. Instead, its main value lies in avoiding slight negative transfer from unsuitable classes while preserving the gains on more responsive ones. This observation is consistent with the object-wise comparison in Table 3, where the main benefits of the proposed method are concentrated on categories such as capsules and especially macaroni2. In contrast, further including pcb1 does not provide additional benefit at the final mean level. We also tested replacing pcb1 with pcb2 in the three-class activation setting, obtaining 95.11 ± 0.44 I-AUC, 98.92 ± 0.01 P-AUC, and 93.96 ± 0.16 PRO. This result is close to A3 and does not provide a clear advantage over the more selective two-class configuration. Overall, these results suggest that selective refinement should be applied to a suitable subset of weak classes rather than uniformly expanded to all candidate difficult categories.

#### 4.5.2. Sensitivity to the Patch Gate Quantile

We further evaluate the sensitivity of the patch gate quantile in Table 6. The results obtained with different quantiles are very close across all three metrics, indicating that the performance of TSMR is not critically dependent on delicate tuning of this parameter. We use 0.10 as the default setting because it gives the best image-level AUROC in the main evaluation, while the other tested values remain within a very similar performance range.

This insensitivity is useful for two reasons. Firstly, it suggests that the observed performance is not driven by delicate tuning of the patch gate quantile. Secondly, it indicates that after the weak-class subset is properly selected, the gating behavior remains stable within the tested quantile range. In other words, the proposed method does not rely on a narrow operating point of this hyperparameter under the final configuration.

#### 4.5.3. Memory and Time Cost

Finally, we analyze the computational cost of the proposed online memory setting by varying $N_{I,\max}$, which controls the maximum number of stored memory items. As reported in Table 7, increasing $N_{I,\max}$ leads to higher GPU memory consumption, while the inference time remains nearly unchanged within the evaluated range. Specifically, the GPU memory increases from 2579 MB to 6117 MB when $N_{I,\max}$ grows from 50 to 1000, whereas the computation time stays at around 64 ms.

These results suggest that, under our current implementation and RTX 3090 hardware setting, increasing the online memory size mainly affects GPU memory usage within the evaluated range, while introducing only a negligible change in average inference time. Therefore, GPU memory is the more direct constraint when using a larger online memory in our experiments. Nevertheless, computation time remains critical for real-time industrial inspection, and our conclusion should not be interpreted as memory size having no effect on latency under unlimited scaling. In practical deployment, the online memory size should be selected by jointly considering the available GPU memory and the latency requirement of the target application.

### 4.6. Qualitative Results

**Figure 3:**
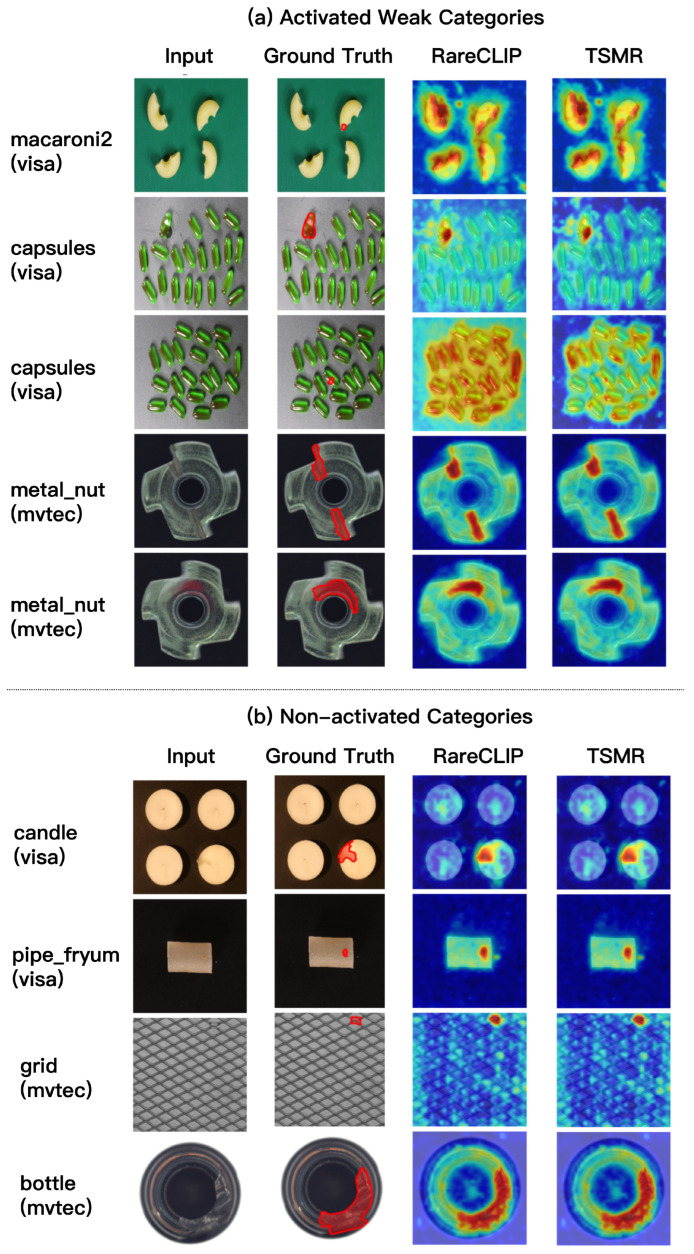
Qualitative comparison on (**a**) activated weak categories and (**b**) non-activated categories.

We further provide qualitative comparisons to examine the anomaly map behavior of TSMR. As shown in Figure 3, the visualization is divided into two labeled parts: activated weak categories in Figure 3a and non-activated categories in Figure 3b. Each row presents the input image, ground-truth mask, RareCLIP anomaly map, and TSMR anomaly map. For activated weak categories, including macaroni2, capsules, and metal_nut, TSMR produces anomaly maps comparable to RareCLIP while suppressing scattered or diffuse responses in selected cases. This is especially visible for capsules, where RareCLIP tends to activate on visually salient normal regions, whereas TSMR gives more selective responses around defective areas. These results are consistent with the goal of TSMR: regulating online memory evolution rather than redesigning the anomaly map generation pipeline.

For the non-activated categories shown in Figure 3b, such as candle, pipe_fryum, grid, and bottle, TSMR largely preserves the behavior of the reproduced RareCLIP baseline. This is expected because these categories are not included in the activated weak-class set, and the proposed selective refinement is not intended to force changes on already stable categories. Therefore, the qualitative results illustrate two aspects of TSMR: it can reduce noisy responses in selected weak-category cases, while maintaining comparable localization behavior on non-activated categories. These observations are consistent with the quantitative results in Table 2, Table 3, and Table 5, where the gains mainly come from selected weak or difficult categories rather than from a uniform improvement across all classes.

Nevertheless, some limitations remain. Since TSMR keeps the RareCLIP anomaly scoring pipeline unchanged, its visual improvements may be moderate in some samples. For categories with extremely subtle defects, strong appearance variation, or ambiguous boundaries, the predicted responses may still be incomplete or may contain residual false activations. TSMR improves the reliability of test-time memory refinement, precise region coverage and boundary delineation remain challenging in difficult industrial scenarios.

## 5. Conclusions

This paper studied online zero-shot anomaly detection for industrial inspection, where target-domain training samples are unavailable during downstream deployment and online memory evolves sequentially from unlabeled test streams. We proposed TSMR, a lightweight extension of the reproduced RareCLIP framework, to improve the reliability of test-time memory evolution without modifying the backbone encoder or redesigning the anomaly scoring pipeline. The core idea of TSMR is to regulate whether the online memory should be refined for the current test frame, rather than applying memory updates uniformly to all incoming samples. Specifically, TSMR integrates a reference confidence quantile gate, a text-prior-based frame consensus gate, and weak-class selective activation to derive a frame-level memory-update decision. This design reduces unreliable memory refinement on visually unstable categories while preserving the original RareCLIP updating behavior for stable categories. Experiments on VisA and MVTec AD showed that TSMR achieves clear improvements on VisA and maintains competitive performance on MVTec AD. Object-wise analysis demonstrated that the benefit of TSMR mainly comes from selected challenging categories, while seed-wise comparison confirmed that the observed improvements remain stable under different online evaluation orders. Ablation studies further verified the importance of weak-class subset selection and showed that the method is robust to a range of patch gate quantiles. Cost analysis indicated that TSMR introduces negligible additional inference time within the evaluated memory range, although larger online memory mainly increases GPU memory consumption. Despite these improvements, the current design still relies on pilot validation to determine the weak-class set and gate hyperparameters, and its gains remain concentrated on selected categories. Future work will investigate adaptive weak-category identification, automatic threshold selection, more robust anomaly map refinement for subtle defects and ambiguous boundaries, and broader validation on stronger zero-shot anomaly detection baselines.

## Figures and Tables

**Table 1:** Main notations used in this paper.

Symbol	Definition
$x\in R^{H\times W\times 3}$	input image
${\left\{f_{i}\right\}}_{i=1}^{N}$	patch-level visual features extracted from *x*
*N*	number of patches in the current image
$M^{\left(t\right)}$	online memory state before processing the current image at step *t*
$M^{\left(t+1\right)}$	online memory state after the frame-level update decision
U(·)	original RareCLIP memory-update operator
*y*	category identity of the current test sample
$\Omega_{w}$	activated weak-class set
$m_{i}$	patch-level reference confidence score of $f_{i}$
${\bar{m}}_{t}$	frame-level mean reference confidence of the current image
H	history set of previous frame-level confidence values
$p_{q}$	quantile parameter for the reference confidence gate
$N_{\min}$	minimum history length required to activate the confidence gate
$G_{\mathrm{cq}}$	frame-level reference confidence quantile gate
${\hat{r}}_{i}$	reference consistency score of patch *i*
$p_{i}^{\mathrm{abn}}$	text-prior abnormal probability of patch *i*
$\tau_{r}$	threshold for the reference consistency condition
$\tau_{t}$	threshold for the text-prior abnormality condition
$a_{i}$	patch-level reliable indicator used for TPB frame consensus
$\rho_{\mathrm{tpb}}$	frame-level reliable ratio computed from ${\left\{a_{i}\right\}}_{i=1}^{N}$
$\tau_{f}$	frame-level fraction threshold for the TPB gate
$G_{\mathrm{tpb}}$	TPB frame consensus gate
$G_{t}^{\mathrm{patch}}$	final frame-level patch-memory update gate
$\Phi_{\mathrm{pix}}\left(\cdot \right)$	baseline anomaly map construction function
$A_{\mathrm{pix}}$	pixel-level anomaly map
$\Phi_{\mathrm{img}}\left(\cdot \right)$	baseline image-level aggregation function
$S_{\mathrm{img}}$	image-level anomaly score

**Table 2:** Comparison with representative zero-shot anomaly detection methods on VisA and MVTec AD. Bold indicates the best result. All metrics are in %. The asterisk (*) denotes a batch/multi-image inference setting.

	Offline						Online			
Metric	WinCLIP [7]	AnomalyCLIP [8]	AdaCLIP [10]	VCP-CLIP [11]	RareCLIP [15]	Ours	Online-InReaCh [26]	MuSc * [14]	RareCLIP [15]	Ours
VisA										
I-AUC	78.1	82.0	85.8	83.8	86.1	**89.0**	78.0 ± 0.2	90.0 ± 0.5	94.4 ± 0.3	**95.1 ± 0.4**
P-AUC	79.6	95.5	95.5	95.7	95.7	**97.1**	95.7 ± 0.1	98.6 ± 0.0	98.8 ± 0.0	**98.9 ± 0.0**
PRO	56.8	86.7	51.3	90.7	90.2	**90.8**	75.7 ± 0.6	92.4 ± 0.1	93.5 ± 0.1	**94.0 ± 0.2**
MVTec AD										
I-AUC	91.8	91.6	90.0	**92.1**	91.5	91.5	87.1 ± 0.9	96.0 ± 0.4	**98.0 ± 0.4**	**98.0 ± 0.4**
P-AUC	85.1	91.1	89.9	**92.0**	91.5	91.5	93.5 ± 0.1	97.0 ± 0.2	97.5 ± 0.4	**97.6 ± 0.5**
PRO	64.6	81.4	44.1	**87.3**	86.2	86.2	83.0 ± 0.4	93.3 ± 0.1	93.5 ± 0.1	**93.6 ± 0.4**

**Table 3:** Object-wise fair comparison between the reproduced RareCLIP baseline and the proposed method on VisA under the online setting. All results are averaged over five seeds.

	I-AUC			P-AUC			PRO		
Object	RareCLIP	Ours	Δ	RareCLIP	Ours	Δ	RareCLIP	Ours	Δ
candle	97.26	97.92	+0.66	99.43	99.44	+0.01	96.27	96.41	+0.14
capsules	92.12	93.66	+1.55	98.35	98.62	+0.27	94.10	95.15	+1.05
cashew	96.96	97.46	+0.50	99.72	99.75	+0.03	95.82	96.38	+0.56
chewinggum	97.99	98.22	+0.24	99.60	99.66	+0.06	94.18	95.12	+0.95
fryum	97.36	97.86	+0.50	97.20	97.73	+0.53	94.11	94.41	+0.30
macaroni1	95.22	95.70	+0.48	99.70	99.77	+0.06	98.04	98.39	+0.36
macaroni2	77.88	79.92	+2.04	98.64	98.73	+0.09	90.28	91.35	+1.07
pcb1	90.40	90.64	+0.24	99.44	99.41	−0.03	92.71	91.52	−1.19
pcb2	94.71	95.66	+0.95	97.88	97.91	+0.03	85.65	86.81	+1.16
pcb3	95.39	96.55	+1.15	97.62	97.87	+0.25	89.99	90.89	+0.91
pcb4	98.71	98.90	+0.19	98.64	98.71	+0.07	92.74	93.41	+0.67
pipe_fryum	98.84	98.76	−0.09	99.32	99.47	+0.15	97.85	97.72	−0.14
mean	94.40	95.10	+0.70	98.80	98.92	+0.13	93.48	93.96	+0.48

**Table 4:** Seed-wise comparison between the reproduced RareCLIP baseline and the proposed method on VisA under the online setting.

	I-AUC			P-AUC			PRO		
Seed	RareCLIP	Ours	Δ	RareCLIP	Ours	Δ	RareCLIP	Ours	Δ
0	94.39	94.92	+0.53	98.80	98.95	+0.15	93.41	93.91	+0.50
1	94.11	94.83	+0.72	98.79	98.91	+0.12	93.40	93.98	+0.58
2	94.56	95.62	+1.06	98.81	98.92	+0.11	93.61	94.08	+0.47
3	94.93	95.53	+0.60	98.80	98.93	+0.13	93.69	94.12	+0.43
4	94.02	94.62	+0.60	98.77	98.91	+0.14	93.29	93.73	+0.44
mean	94.40	95.10	+0.70	98.79	98.92	+0.13	93.48	93.96	+0.48

**Table 5:** Ablation study on weak-class subset selection. A checkmark indicates that the corresponding weak class is activated. Bold indicates the best result.

Activated Weak Classes					Metrics		
Variant	Setting	Cap.	Mac2	PCB1	I-AUC	P-AUC	PRO
A0	Reference				95.08 ± 0.47	**98.92 ± 0.02**	**93.97 ± 0.19**
A1	Cap. + Mac2 + PCB1	✓	✓	✓	95.06 ± 0.47	98.92 ± 0.01	93.96 ± 0.17
A2	Cap. only	✓			95.08 ± 0.46	98.92 ± 0.01	93.97 ± 0.17
A3	Cap. + Mac2	✓	✓		**95.10 ± 0.45**	**98.92 ± 0.02**	93.96 ± 0.16

**Table 6:** Sensitivity analysis of the patch gate quantile. Bold indicates the best result.

PQ	I-AUC (%)	P-AUC (%)	PRO (%)
0.10	**95.10 ± 0.45**	**98.92 ± 0.02**	93.96 ± 0.16
0.11	95.09 ± 0.44	**98.92 ± 0.02**	**93.97 ± 0.16**
0.12	95.09 ± 0.44	**98.92 ± 0.02**	93.96 ± 0.17
0.14	95.09 ± 0.46	98.92 ± 0.01	93.96 ± 0.18

**Table 7:** Memory and time cost under different memory sizes.

$N_{I,\max}$	GPU (MB)	Time (ms)
50	2579	64.2
200	4339	64.4
1000	6117	64.5

## Data Availability

The datasets analyzed in this study are publicly available. MVTec AD is available at https://www.mvtec.com/company/research/datasets/mvtec-ad (accessed on 31 May 2026). VisA is available at https://github.com/amazon-science/spot-diff (accessed on 31 May 2026). The code and additional results are available from the corresponding author upon reasonable request.
